# The role of radiotherapy in the management of malignant peripheral nerve sheath tumors: a single-center retrospective cohort study

**DOI:** 10.1007/s00432-023-05449-9

**Published:** 2023-11-04

**Authors:** Siyer Roohani, Noa Marie Claßen, Felix Ehret, Armin Jarosch, Tomasz Dziodzio, Anne Flörcken, Sven Märdian, Daniel Zips, David Kaul

**Affiliations:** 1 Charité - Universitätsmedizin Berlin, Corporate Member of Freie Universität Berlin and Humboldt-Universität zu Berlin, Department of Radiation Oncology, Augustenburger Platz 1, 13353 Berlin, Germany; 2 Berlin Institute of Health at Charité - Universitätsmedizin Berlin, BIH Biomedical Innovation Academy, BIH Charité (Junior) Clinician Scientist Program, Charitéplatz 1, 10117 Berlin, Germany; 3 Charité − Universitätsmedizin Berlin, Berlin, Germany, German Cancer Consortium (DKTK), partner site Berlin, and German Cancer Research Center (DKFZ), Heidelberg, Germany; 4 Charité – Universitätsmedizin Berlin, Corporate Member of Freie Universität Berlin and Humboldt-Universität zu Berlin, Institute of Pathology, Berlin, Charitéplatz 1, 10117 Germany; 5 Charité – Universitätsmedizin Berlin, Corporate Member of Freie Universität Berlin and Humboldt-Universität zu Berlin, Department of Surgery, Berlin, Germany; 6 Charité − Universitätsmedizin Berlin, Corporate Member of Freie Universität Berlin and Humboldt Universität zu Berlin, Department of Hematology, Oncology and Tumor Immunology, Berlin, Augustenburger Platz 1, 13353 Germany; 7 Charité – Universitätsmedizin Berlin, Corporate Member of Freie Universität Berlin and Humboldt-Universität zu Berlin, Center for Musculoskeletal Surgery, Augustenburger Platz 1, 13353 Berlin, Germany

**Keywords:** Malignant peripheral nerve sheath tumor, MPNST, Radiotherapy, Local control, Survival, Prognostic factor

## Abstract

**Purpose:**

This study sought to investigate the role of radiotherapy (RT) in addition to surgery for oncological outcomes in patients with malignant peripheral nerve sheath tumors (MPNST).

**Methods:**

In this single-center, retrospective cohort study, histopathologically confirmed MPNST were analyzed. Local control (LC), overall survival (OS), and distant metastasis-free survival (DMFS) were assessed using the Kaplan–Meier estimator. Multivariable Cox regression analysis was performed to identify factors associated with LC, OS, and DMFS.

**Results:**

We included 57 patients with a median follow-up of 20.0 months. Most MPNSTs were located deeply (87.5%), were larger than 5 cm (55.8%), and had high-grade histology (78.7%). Seventeen patients received surgery only, and 25 patients received surgery and pre- or postoperative RT. Median LC, OS, and DMFS after surgery only were 8.7, 25.5, and 22.0 months; after surgery with RT, the median LC was not reached, while the median OS and DMFS were 111.5 and 69.9 months. Multivariable Cox regression of LC revealed a negative influence of patients presenting with local disease recurrence compared to patients presenting with an initial primary diagnosis of localized MPNST (hazard ratio: 8.86, *p* = 0.003).

**Conclusions:**

The addition of RT to wide surgical excision appears to have a beneficial effect on LC. Local disease recurrence at presentation is an adverse prognostic factor for developing subsequent local recurrences. Future clinical and translational studies are warranted to identify molecular targets and find effective perioperative combination therapies with RT to improve patient outcomes.

**Supplementary Information:**

The online version contains supplementary material available at 10.1007/s00432-023-05449-9.

## Introduction

Malignant peripheral nerve sheath tumors (MPNST) are malignant spindle cell tumors sporadically arising from peripheral nerves, a pre-existing benign nerve sheath tumor, after radiation exposure or in patients with neurofibromatosis type 1 (NF1) LaFemina et al. 2013; WHO Classification of Tumours Editorial Board 2020). MPNST account for 3–5% of all soft-tissue sarcomas (WHO Classification of Tumours Editorial Board 2020). Typically, 20- to 50-year-old patients present with an enlarging painful or painless mass most commonly located in the trunk or the extremities (WHO Classification of Tumours Editorial Board 2020). MPNSTs are aggressive tumors with an unfavorable prognosis and a high risk of distant metastatic spread (WHO Classification of Tumours Editorial Board 2020; Callegaro et al. 2016; Valentin et al. 2016; Anghileri et al. 2006). Adverse prognostic factors are truncal location, tumor size > 5 cm, local disease recurrence at presentation, high-grade histology according to the Fédération Nationale des Centres de Lutte Contre le Cancer classification, NF1-associated MPNST, radiation-induced MPNST, and heterologous rhabdomyoblastic differentiation (triton tumor) (LaFemina et al. 2013; WHO Classification of Tumours Editorial Board 2020; Guellec et al. 2016; Miao et al. 2019). Multiple retrospective studies confirmed wide surgical excision with clear margins as an essential positive prognostic factor for local control (LC) and overall survival (OS) (Valentin et al. 2016; Miao et al. 2019; Stucky et al. 2012; Dunn et al. 2013). The role of pre- or postoperative radiotherapy (RT), however, remains unclear. This study aims to analyze prognostic factors for the oncological outcomes and the role of RT in addition to surgery in a single-center cohort of MPNST patients.

## Methods

This retrospective, single-center cohort study included adult patients with the histopathologically confirmed diagnosis of MPNST, who received treatment at our institution between 1997 and 2023. We included patients presenting with primary diagnosed, locally recurrent, metastatic or metastatic recurrent MPNSTs. We excluded patients below 18 years of age. We reviewed data on the patient characteristics, imaging, pathology, surgical, oncological, and RT treatment characteristics, and oncological outcome data. Endpoints included LC, OS, and distant metastasis-free survival (DMFS). LC was defined as an unchanged or decreased MPNST volume after surgical excision or last RT treatment (if not resected) or last chemotherapy cycle (if not resected or irradiated) assessed by a board-certified radiologist on follow-up imaging with magnetic resonance imaging (MRI) or computed tomography (CT). OS was defined as the time from primary diagnosis to death by any cause. DMFS was defined as the time from surgical excision or last RT treatment (if not resected) or last chemotherapy cycle (if not resected or irradiated) to radiographic or histopathological evidence of distant metastasis or death by any cause. Radiographic follow-up was calculated from the day of initial therapy until the last available CT or MRI. Clinical follow-up was calculated from the date of initial therapy until the last clinical visit. Patients were censored at the last available follow-up if no local recurrence, death, or distant metastases were observed.

For descriptive statistics, ranges, medians, interquartile ranges, and means for continuous variables were used. LC, OS, and DMFS were assessed using the Kaplan–Meier estimator. Multivariable Cox regression was performed to analyze factors associated with LC, OS, and DMFS. A *p* value of ≤ 0.05 was considered statistically significant. The proportional hazards assumption was tested with a global test using Schoenfeld residuals. Statistical analysis was performed with GraphPad Prism v.9.3.1 (GraphPad Software, San Diego, CA, USA) and STATA MP 16.0 (StataCorp, College Station, TX, USA). Figures were created with GraphPad Prism v.9.3.1 (GraphPad Software, San Diego, CA, USA). The study was approved by the institutional review board (EA1/072/23).

## Results

### Patient and treatment characteristics

The entire cohort comprised 57 patients, of which 17 received surgery alone, 25 received surgery and RT, and 15 patients received other therapies (RT alone, chemotherapy alone, palliative care, etc.). Patient and treatment characteristics are summarized in Table 1. The median age at primary diagnosis was 48 years (range 18–86 years), with more female than male patients (57.9% vs. 42.1%, respectively); patients in the surgery only arm were younger than in the surgery with RT arm (median age 39 years vs. 51 years, respectively). The majority of patients (80.7%) were treated between 2010 and 2023. The most common primary locations were the head and neck area (26.3%), followed by the extremities (22.8%), trunk wall (15.8%), and other locations (35.1%). More than half of tumors (55.8%) had a maximum diameter larger than 5 cm. The proportion of tumors greater than 5 cm was larger in the surgery only group compared to the patients who received surgery and RT (81.2% vs. 72%, respectively). Most MPNSTs were located deeply (87.5% in the entire cohort) and were similarly distributed in both treatment groups. More than half of tumors in the surgery and RT group had grade 3 histology, while the majority of tumors in the surgery only group were grade 2. At initial presentation, most patients (70.2%) had the primary diagnosis of a localized MPNST. Locally recurrent tumors represented 14% of the entire cohort and were similarly distributed between the surgery only and the surgery with RT group. Nine patients with synchronous metastatic disease at presentation received palliative systemic therapy or palliative RT only. NF1-associated MPNST were present in 15 patients (26.3%) in the entire cohort, and the proportion that received surgery only was higher (23.5%) than the proportion in the surgery with RT group (12%). Six cases (10.5%) of all MPNST were associated with prior radiation exposure. The median time between radiation exposure and the development of MPNST was 13.9 years. Two of these patients were treated with surgery only, and three with surgery and RT. One patient in the entire cohort had a heterologous rhabdomyoblastic differentiation (triton tumor) for which he received surgery with RT.

**Table 1:** Patient and treatment characteristics

	All (*N* = 57)		Surgery (*N* = 17)		Surgery + RT (*N* = 25)	
Characteristics	*N*	%	*N*	%	*N*	%
Median age in years (range)	48.0 (18–86)		39.0 (21–69)		51.0 (18–78)	
Sex						
Female	33	57.9	10	58.8	11	44.0
Male	24	42.1	7	41.2	14	56.0
Site						
Trunk wall	9	15.8	3	17.6	4	16.0
Extremity	13	22.8	3	17.6	6	24.0
Head and neck	15	26.3	3	17.6	8	32.0
Thoracic	6	10.5	0	0.0	3	12.0
Retroperitoneum	3	5.3	3	17.6	0	0.0
Abdominal	5	8.8	1	5.9	2	8.0
Spinal	6	10.5	4	23.5	2	8.0
Size						
≤ 5 cm	23	44.2	3	18.8	17	68.0
> 5 cm	29	55.8	13	81.2	8	32.0
N/A	5	–	1	–	0	–
Location						
Deep	49	87.5	15	88.2	19	79.2
Superficial	7	12.5	2	11.8	5	20.8
N/A	1	–	0	–	1	–
Grade (FNCLCC)						
G1	6	15.4	2	15.4	2	9.5
G2	17	43.6	6	46.2	8	38.1
G3	16	41.0	5	38.4	11	52.4
N/A	18	–	4	–	4	–
Presentation status						
Localized, primary diagnosis	40	70.2	12	70.6	20	80.0
Localized, recurrent disease	8	14.0	3	17.6	5	20.0
Metastatic, primary diagnosis	9	15.8	2	11.8	0	0.0
NF1-associated MPNST	15	26.3	6	35.3	6	24.0
Radiation-induced MPNST	6	10.5	2	11.8	3	12.0
Median time in years between radiation and primary diagnosis MPNST (range)	13.9 (5.6–25.4)		16.7 (11.2–22.2)		16.0 (11.8–25.4)	
Triton tumor	1	1.8	0	0.0	1	100
Radiotherapy	27	47.4	0	0.0	25	100
Preoperative radiotherapy	7	25.9	0	0.0	7	28.0
Postoperative radiotherapy	17	63.0	0	0.0	17	68.0
Radiotherapy only	2	11.1	0	0.0	0	0.0
Radiotherapy timing N/A	1	3.6	0	0.0	1	4.0
Median dose per fraction (range)	2.0 Gy (1.8–15)		–		2.0 Gy (1.8–3.8)	
Median total dose (range)	60.0 Gy (49.4–70)		–		60.0 Gy (49.4–66 Gy)	
Chemotherapy (anthracycline-based)	24	42.1	9	52.9	9	36.0
Preoperative chemotherapy	11	45.8	5	55.6	6	66.7
Postoperative chemotherapy	6	25.0	4	44.4	2	22.2
Concurrent radiochemotherapy	1	4.2	0	0.0	1	11.1
Chemotherapy only	6	25.0	0	0.0	0	0.0
RHT	4	7.0	0	0.0	3	12.0
Preoperative RHT and Chemotherapy	2	50.0	0	0.0	2	66.7
Postoperative RHT and Chemotherapy	1	25.0	0	0.0	1	33.3
Chemotherapy and RHT only	1	25.0	0	0.0	0	0.0

*FNCLCC* Fédération Nationale des Centres de Lutte Contre le Cancer, *MPNST* Malignant peripheral nerve sheath tumor, *N/A* not available, *NF1* neurofibromatosis type 1, *RHT* regional hyperthermia, *RT* radiotherapy

RT was mostly delivered postoperatively, with a median dose of 2 Gy per fraction and a total median dose of 60.0 Gy. Twenty-four patients received anthracycline-based chemotherapy, nine in the surgery only group and nine in the surgery with RT group. Four patients received regional hyperthermia and chemotherapy, three combined with surgery and RT, one without surgery or RT.

### Oncological outcomes

Oncological outcomes are summarized in Table 2. The median clinical follow-up in the entire cohort was 20 months, with longer follow-up periods in the surgery with RT group (53.8 months) compared to the surgery only group (16.7 months). The median radiographic follow-up was 18.0 months.

**Table 2:** Oncological outcomes

	All (*n* = 57)	Surgery only (*n* = 17)	Surgery + RT (*n* = 25)
Median follow-up, months (IQR)	20.0 (46.8)	16.7 (9.8)	53.8 (78.1)
Median LC, months	Not reached	8.7	Not reached
Resection margin	*n* = 33	*n* = 14	*n* = 19
R0 (%)	78.8	64.3	89.5
R1 (%)	12.1	21.4	5.3
R2 (%)	9.1	14.3	5.3
N/A (*n*)	9	3	6
Median OS, months	56.9	25.5	111.5
Median DMFS, months	35.9	22.0	69.9

**Fig. 1:**
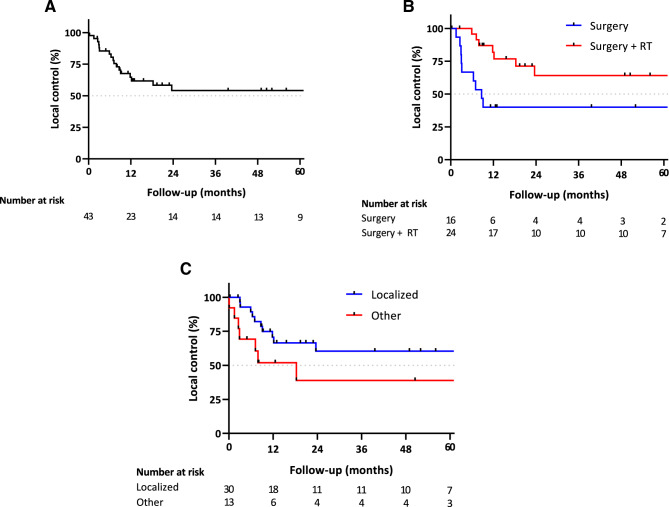
**A** Local control in all patients with available follow-up data. **B** Local control between surgery alone (Surgery) and surgery with radiotherapy (Surgery + RT). **C** Local control between primary localized disease at presentation (Localized) and locally recurrent or metastatic disease at presentation (Other)

Data on local disease control were available in 43 out of 57 patients. In all patients with available follow-up data on local disease control, median LC was not reached (Fig. 1A). Two patients were treated with RT only and one patient with chemotherapy only. In the remaining 40 patients, LC was higher in the patients treated with surgery and RT than in the surgery only group, with a median LC of 8.7 months in the surgery only group and not reached in the surgery with RT group (Fig. 1B). Patients initially presenting with localized disease had longer LC times than patients presenting with local disease recurrence or metastatic disease (not reached in localized disease vs. 18.3 months in locally recurrent or metastatic disease at presentation, Fig. 1C). Additionally, locally recurrent disease vs. localized disease at initial presentation was significantly associated with poorer LC in the multivariable Cox regression (hazard ratio: 8.86, *p* = 0.003, Table 3). The rate of clear surgical margins was higher in the surgery with RT group than in the surgery only group (89.5% vs. 64.3%, respectively).

**Table 3:** Multivariable Cox proportional hazards model for local control

	Multivariable Cox proportional hazards model		
Variable	Hazard ratio	Confidence interval (95%)	*p* value
Treatment			
Surgery alone	Reference		
Surgery + RT	0.42	0.09–2.00	0.276
Size			
≤ 5 cm	Reference		
> 5 cm	0.56	0.1–3.22	0.52
Surgical margin			
R0	Reference		
R1	3.8	0.88–16.42	0.07
Presentation status			
Localized primary diagnosis	Reference		
Locally recurrent	8.86	2.13–36.8	0.003
Grade			
Low-grade	Reference		
High-grade	1.92	0.16–23.45	0.61
Unknown	0.42	0.02–9.32	0.59

*DMFS* distant metastasis-free survival, *IQR* interquartile range, *LC* local control, *N/A* not available, *OS* overall survival, *RT* radiotherapy

**Fig. 2:**
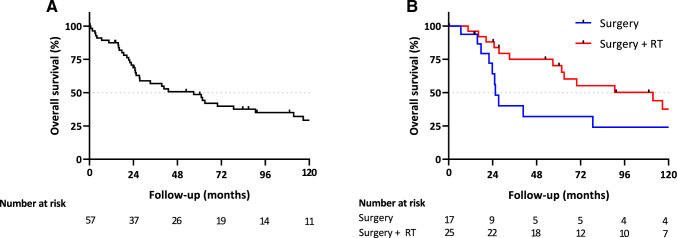
**A** Overall survival in the entire study cohort. **B** Overall survival between surgery alone (Surgery) and surgery with radiotherapy (Surgery + RT)

**Fig. 3:**
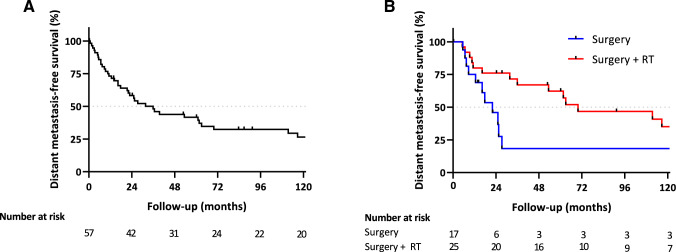
**A** Distant metastasis-free survival in the entire study cohort. **B** Distant metastasis-free survival between surgery alone (Surgery) and surgery with radiotherapy (Surgery + RT)

The median OS in the entire cohort was 56.9 months (Fig. 2A). Between the surgery only and the surgery with RT group, OS curves showed diverging trends with a median OS of 25.5 months in the surgery only group and 111.5 months in the surgery with RT group (Fig. 2B). No significant prognostic factors for OS were found in the multivariable Cox regression (supplementary Table 1). The median DMFS for the entire cohort 35.9 months (Fig. 3A). Similar to the diverging trends in OS, the surgery with RT group also showed longer median DMFS compared to the surgery only group (69.9 months vs. 22 months, Fig. 3B). The multivariable Cox regression analysis did not detect prognostic factors for DMFS (supplementary Table 2).

## Discussion:

Herein, we report our single-institutional retrospective cohort study on 57 MPNST patients. The combination of surgery and RT showed favorable trends in LC over surgery alone and appears to have a beneficial effect on LC. Locally recurrent disease at presentation was a significant adverse prognostic factor for developing subsequent local disease recurrences.

Our findings support previous evidence showing positive effects of surgery and RT for local disease control. A comprehensive retrospective study overlooking 280 patients from the Massachusetts General Hospital by Miao et al. confirmed that pre- or postoperative RT was a significant positive prognostic factor for LC (Miao et al. 2019). Notably, in the present study, the rate of positive surgical margins, as an established risk factor for local recurrences, was higher in the surgery with RT group, than in the surgery only group (WHO Classification of Tumours Editorial Board 2020; Anghileri et al. 2006; Cai et al. 2020; Sobczuk et al. 2020; Martin et al. 2020). An important factor associated with LC in the present study was the presentation status of patients. Initial presentation with locally recurrent disease was a significant adverse prognostic factor for LC in the multivariable regression analysis. Our findings confirm the previous literature describing local disease recurrence at presentation as a risk factor for subsequent relapses and mortality (WHO Classification of Tumours Editorial Board 2020).

Whether an improvement in LC by the addition of RT translates into an OS benefit remains controversial. In a large national database study from the Netherlands comprising 333 not irradiated and 261 irradiated patients, the multivariable Cox regression did not find RT to be a significant prognostic factor for OS (Anghileri et al. 2006; Martin et al. 2020). Similarly, RT was not a positive prognostic factor for OS in the analysis of 353 patients from the French Sarcoma Group and 239 patients from the Warsaw sarcoma center (Valentin et al. 2016; Sobczuk et al. 2020). In contrast to that, RT was a significant positive prognostic factor for disease-specific mortality in the Italian national cancer institute analysis of 205 MPNST patients (Anghileri et al. 2006). Moreover, a comprehensive meta-analysis on prognostic factors for MPNST found a number of studies showing OS benefits by RT (HR: 0.65, *p* = 0.005) (Anghileri et al. 2006; Cai et al. 2020; Fan et al. 2014; Yuan et al. 2017). In our study cohort, an initial trend in OS benefit was also visible in the RT with surgery group (Fig. 2B). However, the multivariable Cox regression analysis could did not confirm this hypothesis (supplementary Table 1). In addition, the proportion of tumors greater than 5 cm in maximum diameter was higher in the surgery with RT group and is an established adverse prognostic factor for survival outcomes (Cai et al. 2020; Martin et al. 2020; Longhi et al. 2010; Mowery and Clayburgh 2019). The distribution of disease sites in the present cohort is unusual. MPNSTs of the head and neck were the most common, while most literature describe the extremities and the trunk as the most common disease sites (WHO Classification of Tumours Editorial Board 2020; Guellec et al. 2016; Ducatman et al. 1986). In the present study, more head and neck MPNST received surgery and RT and this tumor location is known to have a poorer prognosis than MPNSTs of the extremities (Anghileri et al. 2006; Cai et al. 2020; Fan et al. 2014; Yuan et al. 2017).

The present findings on RT for DMFS were comparable to the present results on OS. The median DMFS of surgery with RT was 69.9 months vs. 22 months with surgery only; however, it did not prove to be a significant prognostic factor in the multivariable Cox regression analysis. The Italian MPNST study could not find a DMFS benefit by RT either (Anghileri et al. 2006). In the study from Warsaw, perioperative RT was significantly associated with a negative DMFS outcome (HR: 2.08, p = 0.026) (Sobczuk et al. 2020). Six patients (10.5%) in our study developed radiation-induced MPNST after a median time of 13.9 years after radiation exposure. These findings correlate well with the previous literature where an average latency of 13.5 years between RT and the development of an MPNST is described (Yamanaka and Hayano 2017). Moreover, 15 (26.3%) NF1-induced MPNST were found in the present study. Although no firm conclusions can be drawn from our small sample size, radiation-induced and NF1-induced MPNST are known to carry unfavorable prognoses compared to sporadic MPNST (Miao et al. 2019; Yamanaka and Hayano 2017).

For unresectable and metastasized MPNST, doxorubicin remains first-line chemotherapy, although larger retrospective studies suggest improvements in oncological outcomes by combining ifosfamide with doxorubicin (Kroep et al. 2011; Higham et al. 2017; Yao et al. 2023). Multiple preclinical studies on cell clines and murine models identified molecular targets for MPNST such as EGF and the mTOR signaling pathway with effective in vitro responses to targeted antagonization (Li et al. 2002; Johansson et al. 2008; Endo et al. 2013). Unfortunately, subsequent prospective clinical trials failed to demonstrate clinically relevant responses to targeted therapies (Albritton et al. 2006; Widemann et al. 2016, 2019). MEK inhibitors also displayed preclinical antitumor activity and prospective clinical studies as well as case studies found promising responses, particularly in patients with NF1-associated plexiform neurofibromatosis, a precancerous lesion for MPNST (Gross et al. 2018; Vaassen et al. 2019; Nagabushan et al. 2021; Peacock et al. 2018). The ongoing SARC031 clinical trial (NCT03433183) combines the MEK inhibitor Selumetinib with the mTOR inhibitor Sirolimus in unresectable or metastasized MPNST patients and is expected to complete completion in the near future (Sarcoma Alliance for Research through C, United States Department of D, AstraZeneca 2023). Our sample size of patients receiving systemic therapy for metastasized MPNST only is too small to draw firm conclusions. Thus far, no data are available on effective combination therapies of RT with targeted therapies functioning as radiosensitizers for MPNST, although the synergistic effects on tumor control have been described for other tumor entities (Willers et al. 2021; Willers and Eke 2020; Coleman et al. 2016). Future studies combining targeted therapies with RT are warranted to investigate potential outcome benefits for patients.

The present study carries the intrinsic limitations of retrospective, single-center cohort studies. Within the long study period of 26 years, many new techniques and improvements in the delivery of RT were introduced, which advanced the efficacy and functional outcomes for STS patients (Roeder 2020; Alektiar et al. 2008; Leachman and Galloway 2016). Moreover, the imbalances between both groups may introduce bias in detecting and evaluating oncological outcomes. The follow-up times between groups differed remarkably. Patients receiving surgery with RT had fewer positive surgical margins, were older, had smaller lesions, more head and neck MPNSTs, and more G3 graded tumors than in the surgery only group. Additionally, our study also included patients presenting with locally recurrent or metastatic disease.

MPNSTs are aggressive soft-tissue sarcomas carrying unfavorable prognoses. Wide surgical excision remains the cornerstone of effective local therapy (Dunn et al. 2013). The addition of RT appears to have a beneficial effect on LC. Local disease recurrence at presentation is an adverse prognostic factor for developing subsequent local recurrences. Future collaborative clinical and translational studies are warranted to pool larger datasets, identify molecular targets, and find effective perioperative combination therapies with RT to improve outcomes for patients.

### Supplementary Information

Below is the link to the electronic supplementary material.

Supplementary file1 (DOCX 15 KB)

## Data Availability

Data are available on request from the corresponding author.

## Abbreviations

CT: Computed tomography
DMFS: Distant metastasis-free survival
FNCLCC: Fédération Nationale des Centres de Lutte Contre le Cancer
IQR: Interquartile range
LC: Local control
MPNST: Malignant peripheral nerve sheath tumor
MRI: Magnetic resonance imaging
N/A: Not available
NF1: Neurofibromatosis type 1
OS: Overall survival
PFS: Progression-free survival
RHT: Regional hyperthermia
RT: Radiotherapy
