# Supplementary material for: The role of radiotherapy in the management of malignant peripheral nerve sheath tumors: a single-center retrospective cohort study
Source: J Cancer Res Clin Oncol. 2023 Nov 4;149(20):17739–47. doi: 10.1007/s00432-023-05449-9 (PMC10725397; doi:10.1007/s00432-023-05449-9)
Supplement: Supplementary file 1 — Supplementary file1 (DOCX 15 KB) [file 432_2023_5449_MOESM1_ESM.docx]

| **Supplementary table 1**: **Multivariable Cox proportional hazards model for overall survival** | | | |
| --- | --- | --- | --- |
|  | **Multivariable Cox proportional hazards model** | | |
| **Variable** | **Hazard ratio** | **Confidence interval (95%)** | **p-value** |
| Treatment |  |  |  |
| Surgery alone | Reference |  |  |
| Surgery + RT | 0.49 | 0.14 – 1.76 | 0.28 |
| Size |  |  |  |
| ≤ 5cm | Reference |  |  |
| > 5cm | 0.93 | 0.25 - 3.54 | 0.92 |
| Surgical margin |  |  |  |
| R0 | Reference |  |  |
| R1 | 1.03 | 0.24 – 4.38 | 0.97 |
| Presentation status |  |  |  |
| Localized primary diagnosis | Reference |  |  |
| Locally recurrent | 2.26 | 0.69 – 7.38 | 0.18 |
| Site |  |  |  |
| Extremities | Reference |  |  |
| Trunk | 1.29 | 0.35 – 4-77 | 0.70 |
| Head and neck | 1.08 | 0.24 – 4.95 | 0.92 |
| Abbreviations: RT = Radiotherapy | | | |

| **Supplementary table 2**: **Multivariable Cox proportional hazards model for distant metastasis-free survival** | | | |
| --- | --- | --- | --- |
|  | **Multivariable Cox proportional hazards model** | | |
| **Variable** | **Hazard ratio** | **Confidence interval (95%)** | **p-value** |
| Treatment |  |  |  |
| Surgery alone | Reference |  |  |
| Surgery + RT | 0.89 | 0.26 – 3.07 | 0.85 |
| Size |  |  |  |
| ≤ 5cm | Reference |  |  |
| > 5cm | 1.32 | 0.41 – 4.2 | 0.64 |
| Surgical margin |  |  |  |
| R0 | Reference |  |  |
| R1 | 2.02 | 0.46 – 8.87 | 0.35 |
| Presentation status |  |  |  |
| Localized primary diagnosis | Reference |  |  |
| Locally recurrent | 1.59 | 0.49 – 5.11 | 0.44 |
| Grade |  |  |  |
| Low-grade | Reference |  |  |
| High-grade | 0.41 | 0.07 – 2.43 | 0.33 |
| Unknown | 0.44 | 0.06 – 3.08 | 0.41 |
| Abbreviations: RT = Radiotherapy | | | |
